# 

**Published:** 1873-06

**Authors:** 


					The Southern Dental Association.?The fifth annual meeting of
this association will be held in Baltimore, commencing on the
last Tuesday in July next. This promises to be one of the most
interesting meetings ever held, and we trust it will be well at-
tended by the Southern members of the profession. Efforts are
90 Monthly Summary.
now being made to obtain reduced fare on all railroads in con-
nection with the Baltimore and Ohio, due notice of which will
be given.
American Dental Convention.?The nineteenth annual meeting
will be held on the 12th, (second Tuesday) in August next, at
Saratoga Springs, New York. Dr. 0. S. Hurbut is the Secre-
tary of this association, and will publish an order of business in
the next number of Journal.
TO READERS AND CORRESEONDE
Communications intended for publication, and exchange journals ar
should be addressed to the Editor of the American Journal oi
Science, 259 N. Eutaw St., Baltimore, Md. All letters relating to the
of the journal, or containing cash remittances, to be directed to Messrs. |
& Cowman. Articles for publication should be received by the edito
three weeks previous to the first day of the month on which the numberi
it is designee! for them to appear, is issued; ' ^
i^f=EThe American Journal of Dental Science will,contain fifty
reading matter in each number, making a volume of not less than
Six Hundred pages
EXCLUSIVE OF ADVERTISEMENTS.
T 11 E
AMERICAN JOURNAL
OF
DENTAL SCIENCE.
F. J. S. GORGAS, M. D., D.D. S., Editor.
The Journal is issued on the first of each month and contains not less
than fifty pages of reading matter in each number, exclusive of adver-
tisements, making a volume of six hundred pages per annum.
in each number will be found Original Communications, Corres-
pondence, Selected Articles, Monthly Summary, Bibliographical No-
tices and Editorial Matter, and every effort will be exerted to render
the Journal worthy of the patronage of the Dental profession.
A generous co-operation is respectfully solicited from the members
of the profession ; and as the Journal has now a printing office of its
own which materially lessens the cost of its publication, it will be
issued at the reduced subscription price of
$2.50 3Pcr Annum in. Advance.
Communications are respectfully solicited on all subjects pertain-
ing to the practice of dentistry, as well as reports of cases occurring
in dental practice.
RATES OF ADVERTISING.
1 Page.
* "
i "
1 MO.
i
$15 00
10 00
7 00
5 00
2 MO.
$22 50
15 00
11 00
8 00
3 MO.
$30 00
20 00
15 00
11 00
6 MO.
$50 00
30 00
20 00
15 00
12 MO.
$100 00
50 00
30 00
20 00
All original communications designed for publication, works for
review, new instruments for notice, exchanges, &c., should be directed
to the editor, 2o9 N. Eutaw St., Baltimore) Md.
All advertisements and subscriptions should be addressed to
SNOWDEN & COWMAN,
Publishers of the Am. Journal of Dental Science.
86 W. Fayette St. Bet. Charles & Liberty Sts.,
BALTIMORE, Md.

				

